# Using controls to limit false discovery in the era of big data

**DOI:** 10.1186/s12859-018-2356-2

**Published:** 2018-09-14

**Authors:** Matthew M. Parks, Benjamin J. Raphael, Charles E. Lawrence

**Affiliations:** 1 Department of Physiology and Biophysics, Weill Cornell Medicine, 1300 York Ave, New York, NY 10065 USA; 2 Department of Computer Science, Princeton University, 35 Olden Street, Princeton, NJ 08540 USA; 3 Center for Computational Molecular Biology, Brown University, 115 Waterman Street, Providence, RI 02912 USA; 4 Division of Applied Mathematics, Brown University, 182 George Street, Providence, RI 02912 USA

**Keywords:** False discovery rate (FDR), Big data, Hypothesis testing, High dimensional inference

## Abstract

**Background:**

Procedures for controlling the false discovery rate (FDR) are widely applied as a solution to the multiple comparisons problem of high-dimensional statistics. Current FDR-controlling procedures require accurately calculated *p*-values and rely on extrapolation into the unknown and unobserved tails of the null distribution. Both of these intermediate steps are challenging and can compromise the reliability of the results.

**Results:**

We present a general method for controlling the FDR that capitalizes on the large amount of control data often found in big data studies to avoid these frequently problematic intermediate steps. The method utilizes control data to empirically construct the distribution of the test statistic under the null hypothesis and directly compares this distribution to the empirical distribution of the test data. By not relying on *p*-values, our control data-based empirical FDR procedure more closely follows the foundational principles of the scientific method: that inference is drawn by comparing test data to control data. The method is demonstrated through application to a problem in structural genomics.

**Conclusions:**

The method described here provides a general statistical framework for controlling the FDR that is specifically tailored for the big data setting. By relying on empirically constructed distributions and control data, it forgoes potentially problematic modeling steps and extrapolation into the unknown tails of the null distribution. This procedure is broadly applicable insofar as controlled experiments or internal negative controls are available, as is increasingly common in the big data setting.

**Electronic supplementary material:**

The online version of this article (10.1186/s12859-018-2356-2) contains supplementary material, which is available to authorized users.

## Background

Methods based on the false discovery rate (FDR) [1] have emerged as the preferred means to address the multiple comparisons problem of high-dimensional statistical inference and are widely applied across the sciences [2–5]. The crucial component impacting FDR estimates is the unknown shape of the tail of the null distribution [6]. In settings with limited data, many FDR-controlling procedures rely on assumptions about the nature of the tails of the null distribution or build approximations to these tails using subsets of the test data [2, 6, 7]. In these procedures, FDR estimates involve extrapolation into the unobserved tails of the null distribution.

The increasingly common “big data” setting, wherein thousands of data points are obtained for thousands of variables simultaneously, is revolutionizing statistical analysis across disciplines [8] and presents new opportunities for controlling the FDR. In particular, in big data analysis, a wealth of control data is often available, either from separate controlled experiments or from internal negative controls. Control data can be obtained from a broad range of experimental and data collection regimes. A controlled experiment can be a separate protocol in which all environmental and experimental variables match as closely as possible with those of the test experiment except for the treatment applied. Alternatively, internal negative controls may consist of a subset of data points within the test experiment which are a priori determined to be unaffected by the treatment [9, 10]. Control data has been used to improve FDR estimates through improved parametric or non-parametric models. However, we show that the frequently-available large amount of control data in the big data setting permits estimates of the FDR that rely on fewer assumptions and are simpler and more direct. Here we describe an FDR-controlling procedure that dispenses with often complicated intermediate calculations of *p*-values, model adjustments, and extrapolation and instead models the tails of the null distribution directly. This is demonstrated below with kernel density estimation.

Many extant methods assume that the tail behavior of the null distribution can be accurately estimated via extrapolation and rely on an assumed parametric model for this purpose. But these assumptions are difficult to verify, and when misspecified, can compromise the performance of the FDR-controlling procedure [7]. To address these problems, control data has been used for assessing significance and improving FDR estimates in various manners. For instance, some approaches use control data to obtain more accurate *p*-values by estimating the parameters of an assumed parametric distribution for the null [10–16], but subsequent application of an extant FDR-controlling procedure is still subject to model misspecification [7].

Some procedures use control data to obtain more accurate *p*-values from non-parametric methods [17, 18], but continue to rely on extrapolation into the tails of the null distribution through an extant FDR-controlling procedure. Additionally, as FDR estimates are sensitive to small absolute errors in p-value calculations, often excessive non-parametric sampling is necessary to ensure reliability [19]. Further, *p*-values obtained from resampling are often reported incorrectly, further compromising FDR estimates [20]. Thus, while control data in principle permits accurate p-value computation and FDR estimation, in practice the intermediate step of accurately calculating small *p*-values for the entire set of test data is frequently problematic.

Control data has also been used to make more direct estimates of FDR. For instance, an algorithm that makes positive calls is applied to both the test data and the control data, separately, and the FDR is then estimated from a ratio involving positive calls for test and control data [21–24]. While these kinds of methods are non-parametric, empirical, and informed by control data, they return a point estimate of FDR rather than distributions of test statistics and FDR estimates. Thus, they do not yield q-values [25] or local FDR estimates per data point.

Here, we extend direct empirical approaches to describe a general method for empirically estimating both local and global FDR in big data settings by utilizing control data to directly compare the test and control distributions. Our procedure avoids the intermediate step of calculating accurate *p*-values, which is can be challenging and complicated and often compromises the reliability of a subsequently applied FDR controlling procedure [7, 19, 20]. By using control data, as is frequently found in big data studies, we model the tails of the null distribution directly and forgo extrapolation steps common to many extant FDR controlling procedures. The empirical nature of this approach permits us to assume only that the control data is a reliable representation of the experimental variability, rather than having to invoke stronger assumptions about the parametric forms of the distributions and the dependence structure of the observations. Omitting potentially problematic steps in FDR calculations, the simplified method presented here adheres more closely to a core tenet of experimental science: that significance is assessed by directly comparing test data to control data. As the big data revolution continues to expand across and within disciplines, the procedure described here offers a new tool for reliable assessment of statistical significance.

## Methods

### Bayesian formulation of the FDR

We formulate the test data as a finite mixture drawn from unaffected and affected distributions, as is common for FDR-controlling procedures:

1 $$ f(x)=\lambda \bullet {f}_0(x)+\left(1-\lambda \right)\bullet {f}_1(x)\kern0.75em $$

where *f* is the mixture density of the test data; *f*_0_, *f*_1_ are the unaffected and affected densities by treatment of domain-specific processes, respectively; and λ is the mixing proportion, i.e. the *a priori* probability that a data point was drawn under the null hypothesis. Adopting the Bayesian perspective, we determine statistical significance via the posterior,

$$ P\left(x\ \mathrm{is}\ \mathrm{unaffected}\ |\ {x}_1,\dots, {x}_n\right)=\frac{\lambda \bullet {f}_0(x)}{f(x)}\kern0.5em , $$

which is called the local FDR [7].

Often in high-throughput experiments, only a modest subset of the test variables are expected to be affected. Therefore, in practice we approximate the local FDR by the upper bound for the posterior probability that a data point is unaffected,

2 $$ P\left(x\ \mathrm{is}\ \mathrm{unaffected}\ |\ {x}_1,\dots, {x}_n\right)\le \frac{f_0(x)}{f(x)},\kern0.5em $$

as used by Efron [7].

The global FDR is the ratio of the expected number of unaffected observations *N*_*u*_ above a specified critical value *x*_*c*_ of the test statistic, to the expected total number of observations *N*_*t*_ in the test set above *x*_*c*_:

3 $$ \mathrm{FDR}\left({x}_c\right)=\frac{E\left[{N}_u\right]}{E\left[{N}_t\right]}=\frac{n_u\bullet P\left({X}_u\ge {x}_c\right)\ }{n_t\bullet P\left({X}_t\ge {x}_c\right)} $$

where *n*_*u*_, *n*_*t*_ are the observed numbers of unaffected and total data points, respectively, and *X*_*u*_, *X*_*t*_ are random variables denoting the unaffected and total test statistics, respectively. Many extant methods use the number of observations in the treated sample above the critical value to estimate the denominator and extrapolate from a parametric null distribution to obtain an estimate of the numerator. With empirical controls, both the numerator and the denominator of the global FDR can be estimated by counting the number of observations above the critical value for each sample.

### Assumptions

The empirical nature of our method means that we require two assumptions inherent to experimental science:

1. The controls in the study reasonably represent the unaffected data points in the test set. Specifically, the process that generates the unaffected observations within the test data is the same as the stochastic process which generates the control data; that is, the processes contain similar errors, biases, artifacts, etc.
2. The control data is drawn from the unaffected population and does not contain affected data points.

The implication of the first assumption is that the tails of the control and test distributions are qualitatively similar. The contribution of this work is based on the conviction that the tails of the unaffected distribution are better estimated by control samples than by *p*-values obtained from parametric or non-parametric methods, and thus rests on the two assumptions above. In contrast to many extant FDR-controlling procedures, we do not assume a parametric form or dependence structure for the data.

### Algorithm: Control data-based empirical FDR

A general algorithm for our approach is as follows:

1. Define a test statistic *X* appropriate for the application.
2. Empirically construct *f*, the mixture distribution of the test statistic, from the test data.
3. Compute *X*_*c*_, the set of observed test statistics for the control data.
4. Empirically construct *f*_*c*_ from *X*_*c*_.
5. (optional) Identify the modes *m*_*c*_ and *m*_*t*_ of the control and test distributions, respectively. If these modes differ due to technical artifacts such as sampling error or the method of density construction, then construct *f*_0_ from *f*_*c*_ and *f* by translating *f*_*c*_ by γ, where *γ* = *m*_*c*_ − *m*_*t*_ is the difference of the modes of the test and control distributions, respectively. Specifically: *f*_0_(*x*) = *f*_*c*_(*x* + *γ*). Otherwise, set *f*_0_ = *f*_*c*_. Note that if γ is large, then this suggests that the control data does not accurately represent the experimental condition of the test data, and the results may be unreliable.
6. Determine the local FDR via equation (2) or global FDR via equation (3).

This approach is demonstrated below.

## Results

### Application background

Our motivating example is from molecular biology: the problem of identifying regions of the human genome which have been deleted or duplicated via non-allelic homologous recombination (NAHR). NAHR is a common cellular mechanism that causes large rearrangements of the genome by incorrect DNA repair in long, highly similar (homologous) regions of the genome, known as segmental duplications. In brief, pairs of long, homologous loci (each ≥ 1 kb in length, ≥ 90 identity) may recombine during replication or repair, resulting in the deletion, duplication, or inversion of large segments (1 kb to 1 Mb in length) of intervening DNA sequence (reviewed in [26–28]). Because NAHR occurs at highly similar sequences, the number of genomic loci that are susceptible to NAHR is relatively small: only thousands of genomic loci in the human genome of approximately 3 billion nucleotides. All other regions of the human genome are not susceptible to NAHR or are exceedingly unlikely to undergo NAHR since they do not fulfill the stringent homology requirements of NAHR.

In previous work, we developed a Bayesian algorithm for genome-wide detection of NAHR events using high-throughput DNA sequencing data [29]. Our study focused on a subset of *n =* 324 regions susceptible to deletions and duplications via NAHR across 44 human individuals. These regions were obtained from a segmental duplication database [30] of the human genome. An unusually high or low number of reads mapped to a particular NAHR-susceptible region (called read-depth) may indicate the occurrence of a duplication or deletion via NAHR. There are several known sources of bias that affect read distribution [29]. Benjamini & Speed found that an adjustment for guanine/cytosine (GC) content addressed such biases [31], which lead to our choice of a test statistic.

**Fig. 1:**
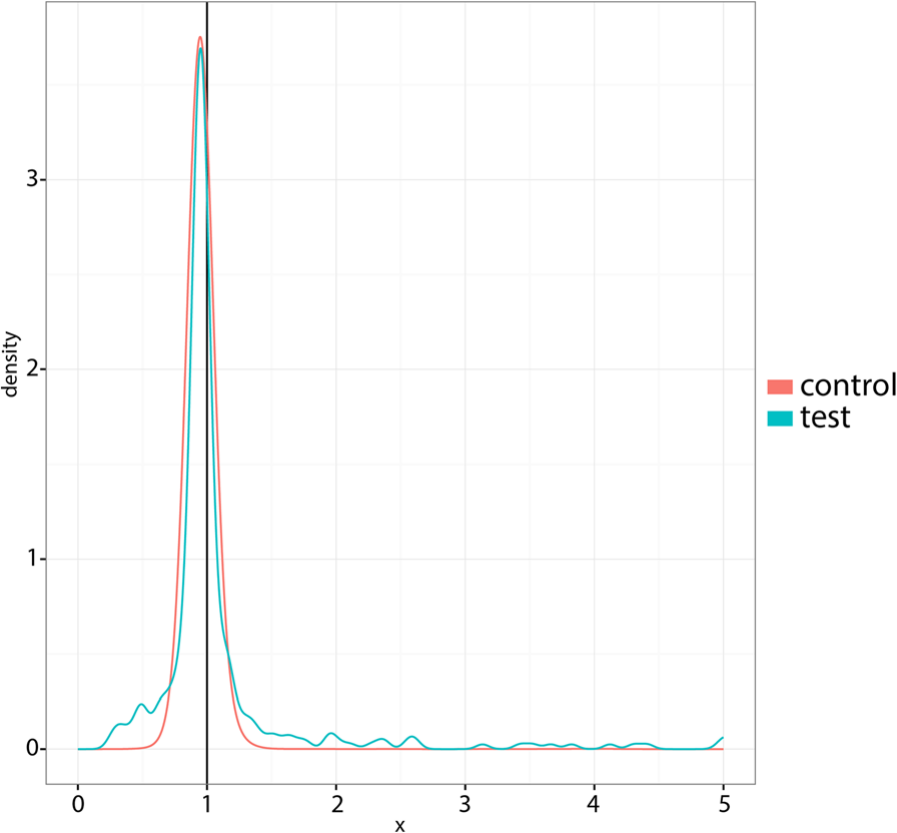
Empirical probability density functions *f* and *f*_*c*_ for the observed read depth ratios for the test and control data, respectively. Both density functions were obtained by kernel density estimation with a Normal kernel. The vertical black line indicates *y* = 1

Our test statistic is the ratio of observed read-depth to mean read-depth for an a priori defined NAHR-susceptible interval of the genome. Namely, the observed read-depth is the number of reads mapped to the given region, and the mean read-depth is the average number of reads mapping to that region, taking into account various sequence composition characteristics known to affect read-depth (see [29, 31]). For a particular genome, the empirical distribution *f* of test data across the *n* = 324 regions that are susceptible to NAHR deletion or duplication is shown in Fig. 1. We expected that only a modest subset of the NAHR-susceptible genomic loci actually experienced an NAHR deletion or duplication.

### Constructing a control distribution from control data

In our big data scenario, with many data points from regions throughout the human genome, we realized that we could empirically construct a control distribution *f*_*c*_ from data known to be drawn from the null hypothesis, and use *f*_*c*_ to derive the null distribution *f*_0_ directly, without further assumptions about the test data.

Because the mechanism of NAHR is well-established [26–28], it is possible to confidently delineate regions of the genome that are not susceptible to rearrangement via NAHR to define a set of internal control regions. Since only a relatively small number of loci in the human genome are susceptible to NAHR, we sampled regions from the large remaining portion of the human genome to obtain internal control data points, or negative controls, and sought to empirically define *f*_*c*_ from these regions. For the purpose of defining the control distribution, negative controls from within the test dataset and data obtained from separate, controlled experiments serve the same purpose.

We randomly sampled 324 ∙ 10 internal control regions for each of the 44 genomes separately, i.e. regions not susceptible to NAHR. The distribution of read-depth across the genome in whole genome sequencing experiments has been extensively studied, and GC content has been found to be the major source of variation in read-depth by genomic region [31–36]. It has been shown that GC content-specific correction factors can be used to remove dependence on GC content in the analysis of read-depth [31]. By employing a test statistic based on GC content-specific correction factors, our test data and control data are expected to follow the same distribution under the null. We performed kernel density smoothing with a Normal kernel to obtain *f*_*c*_ from the control data points. Alternatively, other methods of empirical density construction can be used. Figure 1 shows *f* and *f*_*c*_ for a representative genome analyzed. The control distribution *f*_*c*_ has long tails that are inconsistent with a normal distribution or a mixture of Gaussians. With this large number of controls, there is little advantage in using a parametric control distribution rather than a non-parametric distribution.

### Deriving the null distribution from the control distribution

It is tempting to declare that the unaffected distribution *f*_0_ is equal to the control distribution *f*_*c*_. However, due to artifacts arising from the method of density estimation used to construct the test and control distributions, the modes of the test and control densities may differ slightly and compromise subsequent analysis. For instance, many density estimation methods depend critically on the smoothing parameter, which is often difficult to choose [37]. The principal purpose of using control data is to learn the shape of the tails of the null distribution, which may be difficult to model with a parametric form. At the discretion of the statistician, it may therefore be more conservative, within the context of the specific application, to obtain the null distribution by shifting the control distribution so that the modes of the test data and control data agree. This optional step employs an additional assumption: that most of the test data is drawn from the unaffected distribution, and therefore the mode of the test data is in fact the mode of the unaffected distribution. Nevertheless, this optional step adheres to the purpose of using control data; that is, to inform on the shape of the tails of the null distribution.

For the sake of demonstration, we introduce a location parameter γ and define *f*_0_(*x*) = *f*_*c*_(*x* + *γ*). Under the assumption that most of the test data is drawn from the unaffected distribution, we reason that the mode of the test data is in fact the mode of the unaffected distribution. Thus, γ is the difference in the modes of the control distribution *f*_*c*_ and test distribution *f*. With *m*_*c*_ and *m*_*t*_ being the mode of the control and test distributions, respectively, then *γ* = *m*_*c*_ − *m*_*t*_. We found γ to be consistently small across the 44 individuals analyzed (Additional file 1: Figure S1), consistent with the difference in modes of the control and test distributions arising merely as an artifact of the empirical density construction, and not due to confounding factors affecting the control and test data differentially.

### Results for the NAHR application

We applied our control data-based local FDR procedure to data obtained from the 1000 Genomes Project [38] for 44 human genomes. In particular, for each of the 44 individuals separately, we constructed the empirical null distribution by randomly sampling data from the control regions of the genome, and compared the test data to the control data as outlined in the algorithm above. The test data (Additional file 2: Table S1) was derived from the read counts for a subset of 324 regions of the genome that are a priori susceptible to NAHR rearrangements according to the established mechanistic knowledge of NAHR [26–28, 30], i.e. the same 324 regions for each of the 44 individuals. On the other hand, for each of the 44 individuals, a different set of 3240 control regions were randomly sampled to form the control data (Additional file 3: Table S2).

We found that the numbers of significant calls (local FDR < 0.05) varied modestly among the 44 individuals analyzed in this study (Additional file 4: Figure S2). All of the individuals analyzed are considered to be normal, healthy individuals, so we did in fact a priori expect similar results across individuals. Further, the location adjustment applied to the control distribution to obtain the null distribution was similarly small across all individuals (Additional file 1: Figure S1). Altogether, these similarities across all individuals, despite that the analysis of each individual involved an entirely distinct control dataset, demonstrate the robustness of the procedure to variability in control data.

In our previous work [29], using a local FDR threshold of 0.05, we found that numerous genes are affected by the NAHR-mediated genomic rearrangements, including genes implicated in genetic disorders and with clinical relevance. For example, we called an NAHR deletion on chromosome 5 that deletes gene *GTF2H2*. This gene encodes for a transcription factor and has been linked to spinal muscular atrophy, a common and lethal autosomal recessive neurodegenerative disorder [39, 40].

### Comparison to existing FDR-controlling procedures

#### Assumed parametric forms

A typical statistical approach to address the multiple comparisons problem is the following: (i) specify some parametric model for the test statistic (read-depth of a genomic region in our example) under the null hypothesis, or a non-parametric method; (ii) calculate a *p*-value from this model; (iii) control the FDR by some procedure (e.g. [1, 2, 7]). But posing a parametric model that reliably models the tails of the null distribution of the test statistic, the first step in the approach, is difficult [29, 31, 32]. Non-parametric procedures can avoid extrapolation but require immense computational resources in studies involving more than hundreds of simultaneous tests, and are still subject to model misspecification if the assumptions about how the samples were drawn are incorrect [7].

Extant FDR-controlling procedures assume that accurate *p*-values have been obtained. Indeed, it is likely that in many cases when FDR methodology is applied, the *p*-values were generated from a misspecified model, which has been shown to hinder FDR-controlling procedures [7]. By empirically constructing the null distribution directly from the control data, our strategy relieves the researcher of having to derive accurate p-values. In practice, this will often play to the researcher’s strengths. For instance, following our strategy, an experimental biologist can focus on designing an appropriate controlled experiment or confidently identifying reliable negative controls, rather than attempting to obtain accurate p-values, which may not be the researcher’s area of expertise.

#### Other uses of control data

Control data has been used to empirically estimate the FDR by swapping samples: switching the role of control data and test data, and computing the global FDR as the number of calls made for the control data divided by the number of calls made for the test data [21, 23, 24]. These methods do not specify or use a test statistic, but rather, they calculate the ratio of the number of calls for different thresholds of some parameter, say *θ*, of the algorithm employed. By varying the parameter threshold of the algorithm, a function *h(θ)* for the empirical FDR is obtained. The number of data points whose score *θ* exceeds a given threshold does not define a test statistic because it collapses the data into a single value. As such, in these methods, no null distribution nor test distribution is constructed, and so the local FDR or q-value cannot be computed.

#### Efron’s local FDR

Efron’s local FDR approach [7] attempts to address this model misspecification problem by allowing the null distribution of the inverse standard normal transformed *p*-values to deviate from the theoretical null distribution of *N(0,1)*. Namely, a small portion of the test data around the mode, assumed to be almost entirely drawn from the null, is used to obtain empirical estimates of parameters *μ,σ* to define the null distribution as *N(μ,σ)*. While this is shown to yield improved results over the classical parametric approach, this procedure still has two key assumptions: first, that the correct distribution for the test statistics was employed to obtain accurate *p*-values; and second, that extrapolation of tail values from a selected subset of the p-values is accurate.

We emulated the local FDR approach described by Efron [7] to compare it to the control data-based approach described here. While Efron’s local FDR approach was applied to z-transformed p-values, here we applied the procedure to the test statistic directly. This is appropriate because the genomes analyzed have large numbers of mapped reads, and thus for long regions such as those vulnerable to NAHR events (1 kb to > 100 kb) [30, 41], hundreds to thousands of reads are expected to have been sampled from these regions under the null hypothesis. Further, case studies of the genetic mechanism at hand indicate that the rate of NAHR is relatively low [29], and thus the local FDR assumption that the bulk of the test data is from the null is indeed valid. As such, the central limit theorem’s asymptotic properties apply and it is reasonable to assume that our test statistic is approximately normally distributed, and thus we can apply Efron’s local FDR procedure to the test statistic itself.

**Fig. 2:**
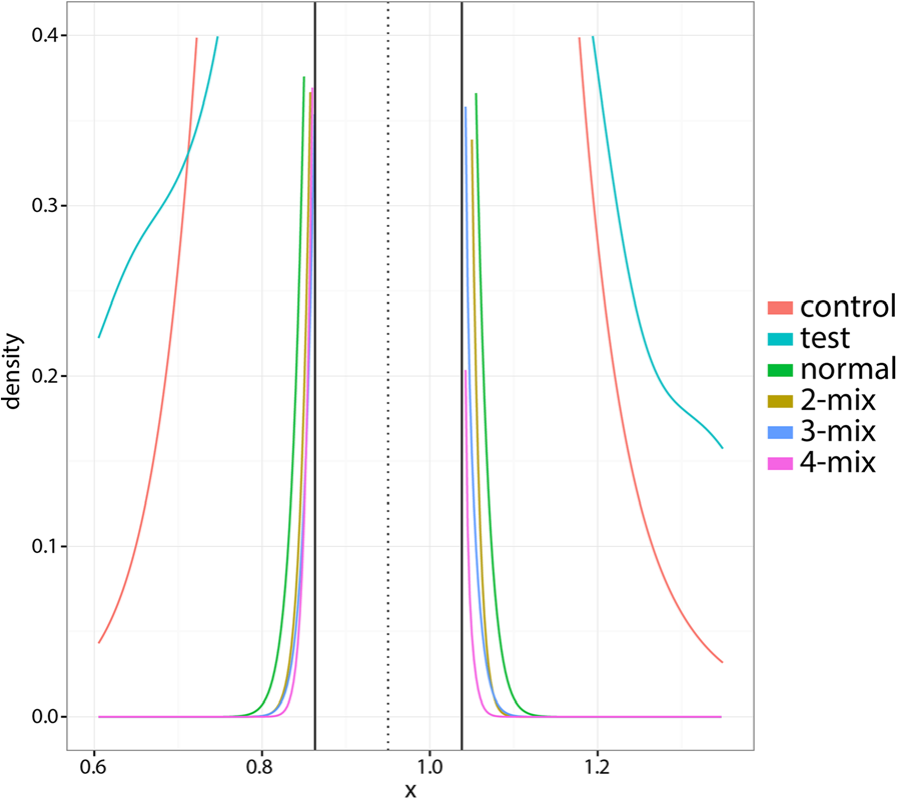
Probability density functions for the test distribution, mode-shifted control distribution, and 1-, 2-, 3-, and 4- component Gaussian mixtures fitted to the central region of the test data. The vertical dotted black line indicates the mode of the test data. The vertical solid black lines indicate the boundaries of the half-height region

Following the local FDR procedure, we defined the half-height region to be the region about the mode of the test distribution where the test density is half of the test density at the mode, i.e. *H* = (*x*, *y*) where $x<m,y>m,f(x)=f(y)=\frac{f(m)}{2}$ and *m* is the mode of the test density *f*. Since a large portion of the test data is expected to be drawn from the null, the half-height region should be composed almost entirely of data points from the null model. We then fit various parametric distributions to the subset of test data points lying within the half-height region. Local FDR values were obtained via equation (2). Our main result, that parametric models poorly fit the tails of the unaffected distribution leading to underestimates of the FDR, also holds for several other distributions (Table 1**;** Fig. 2).

**Table 1:** Number of test data points that are significant (FDR < 0.05) according to various strategies for controlling the FDR. “Control data” indicates the control data-based local FDR strategy described in the present work. All other strategies indicate the assumed parametric form for the null distribution whose parameters are estimated via Efron’s semi-parametric local FDR method. Results are shown for a representative individual

null distribution form	number of significant calls
control data	47
lognormal	106
2-mix	118
3-mix	119
4-mix	123
normal	106

The control data-based approach is more conservative than semi-parametric approaches in the manner of [7]. As shown in Table 1, about half as many tests pass an FDR 0.05 threshold for the control-based approach as under the local FDR approach under several assumed parametric distributions. Indeed, this is because the control-based approach reflects the true tail behavior better than these parametric models (Fig. 2).

The values taken by the null distribution are the focus of FDR-controlling procedures and parametric hypothesis testing in general. The central peak of our control data is similar to the peak of a Gaussian (Fig. 1), but importantly, the tails diverge (Fig. 2). Using control data, we see that in our example such extrapolation would be inaccurate and compromise the reliability of our results.

## Discussion

The complex nature of the data and the large number of comparisons encountered in large-scale, big data studies presents serious challenges for traditional hypothesis testing and *p*-value approaches. In these studies the main challenge is often to distinguish events affected by a treatment from those that are unaffected. The rationale behind the method proposed here is that control datasets in science offer a time-tested means to characterize the behavior of unaffected events. We have outlined a simple method for determining local and global FDR empirically using only control and test data. Because of the empirical nature of our approach and its reliance on only two weak assumptions, it is robust in different settings. These assumptions are sufficiently broad to accommodate the use of control data derived from controlled experiments or negative controls from various experimental protocols. Extant, popular experimental designs amenable to this statistical framework in computational biology include chromatin immunoprecipitation sequencing (ChIP-seq) analyses of DNA-binding factors and RNA-seq analyses of differential gene expression.

The usefulness of our approach depends on the quality of the data. The fundamental assumption of the approach, and indeed of all experimental science, is that the biases, errors, and inherent variation of the experiment are not systematically or selectively different for the control data than for the test data. Our approach is valid to the extent that the control data is qualitatively similar to the test data, and this therefore comprises our assumptions. Therefore, the chosen control dataset is an experimental variable affecting the outcome of the procedure. For instance, in our example, the sampling of internal negative control regions from the unaffected portions of the genome may contribute to variability in the results (Additional file 4:Figure S2). Consequently, verification of the results of our method are not different than with other FDR-controlling procedures.

While this empirical method avoids the requirement that the test statistic follows a specified probability distribution, it does not completely obviate the need to take care in the choice of a test statistic. It remains important to choose a test statistic that neutralizes the impacts of ancillary features that add extraneous noise. From this perspective, the optional location parameter step in our general procedure provides a preliminary measure of the reliability of the control data and robustness of the chosen test statistic. In our example setting, the similarly small location parameter differences across individuals and the similar numbers of significant calls of the procedure across individuals indicate robust results. With this in mind, interpretation of statistical significance according to the FDR produced by our procedure is the same as with other methods, and experimental validation remains an important step for verification of the reliability of the control data and consistency of the experimental regimes analyzed.

In some studies focused on changes, such as changes in gene expression, it is appropriate to use test and matched control experiments to calculate the test statistics to conduct a hypothesis test. Thus, to obtain values of the test statistic for the unaffected population, another set of matched controls is required, yielding comparisons of the within-treatment control samples to the between-treatment control and test differences. While taking this approach may increase the cost of such studies, it provides the only means known to use for avoiding the hazards of misspecification and the mathematical or computational challenge of estimating accurate *p*-values.

This approach relies on two key assumptions of experimental science: that controls are obtained in a manner that reasonably represents the unaffected population, and that the control data does not contain affected data points. It capitalizes on these two assumptions by directly comparing the test and control distributions. In so doing, our approach dispenses with p-values by working directly on the data, rather than relying on the somewhat abstract concepts of statistical hypothesis testing.

## Conclusions

FDR-controlling procedures employed for multiple comparisons problems are a fundamental part of high-dimensional inference and big data analysis, but they often rely on potentially problematic intermediate steps involving modeling assumptions and extrapolation. The statistical framework described here demonstrates a general method for using control data to reliably control the FDR by relying on direct empirical comparisons between test and control data, thereby avoiding complicated intermediate calculations and modeling assumptions that are difficult to verify. As control data from controlled experiments or internal negative controls are a common feature of big data analyses, the procedure presented here demonstrates a shift in statistical paradigm to more closely adhere to the basic tenets of experimental science: that conclusions are drawn from direct comparison of test and control data.

## Additional files

Additional file 1: **Figure S1.** Histogram of the absolute difference γ between the modes of the empirically constructed test and control distributions across the 44 human individuals analyzed. (PNG 34 kb)

Additional file 2: **Table S1.** Test statistics per region and per individual for the test data analyzed in the present work. (TXT 231 kb)

Additional file 3: **Table S2.** Test statistics per region and per individual for the control data analyzed in the present work. (TXT 3328 kb)

Additional file 4: **Figure S2.** Histogram of the number of calls passing local FDR threshold of 0.05 using our control data-based method. (PNG 35 kb)

## Abbreviations

ChIP-seq: Chromatin immunoprecipitation sequencing
DNA: Deoxyribonucleic acid
FDR: False discovery rate
GC: Guanine/cytosine
NAHR: Non-allelic homologous recombination
RNA-seq: Ribonucleic acid sequencing
