# Supplementary figures and images for: Using controls to limit false discovery in the era of big data
Source: BMC Bioinformatics. 2018 Sep 14;19:323. doi: 10.1186/s12859-018-2356-2 (PMC6137876; doi:10.1186/s12859-018-2356-2)

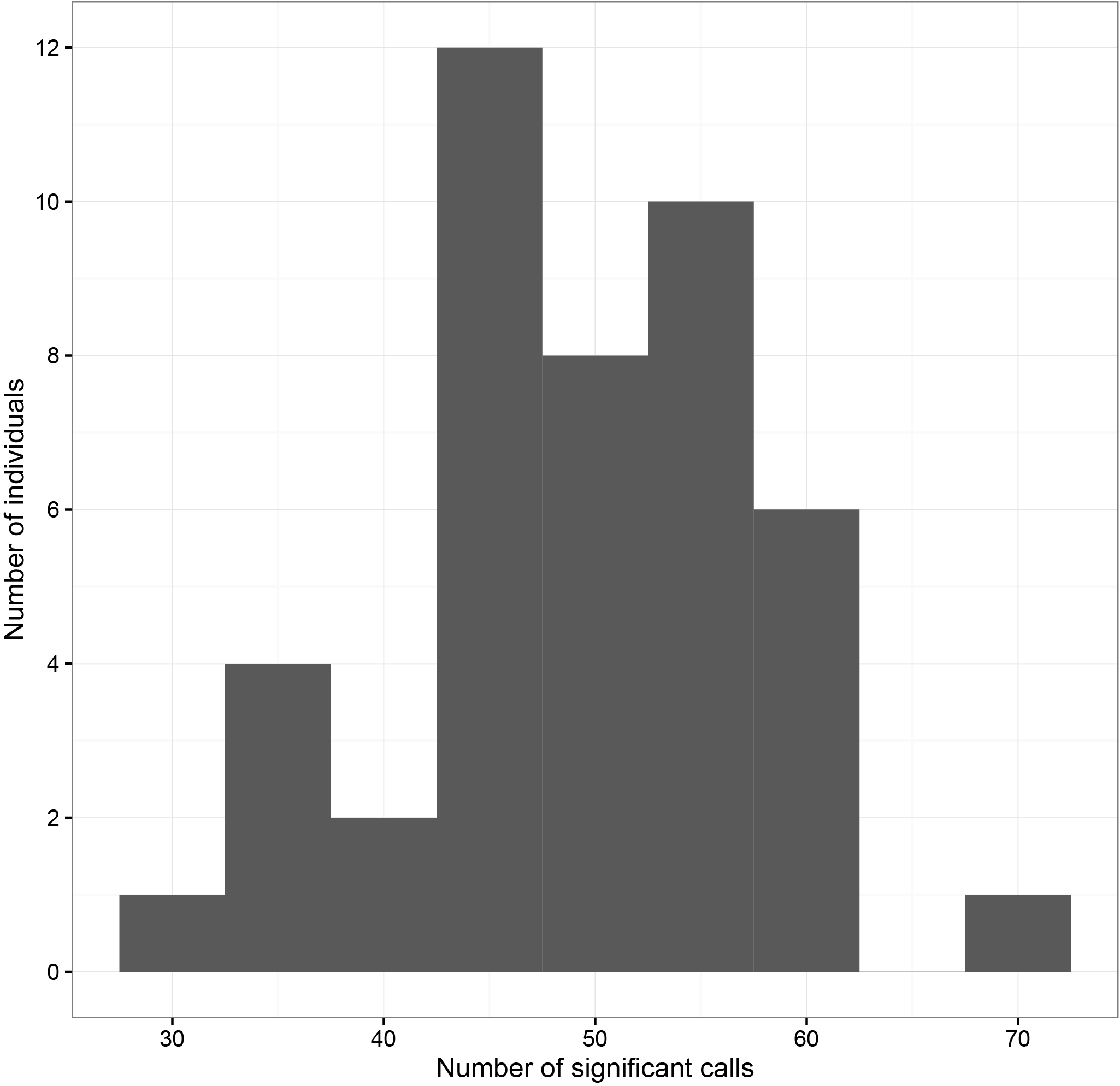

Supplement: Supplementary file 1 — Figure S1. Histogram of the absolute difference γ between the modes of the empirically constructed test and control distributions across the 44 human individuals analyzed. (PNG 34 kb) [file 12859_2018_2356_MOESM1_ESM.png]

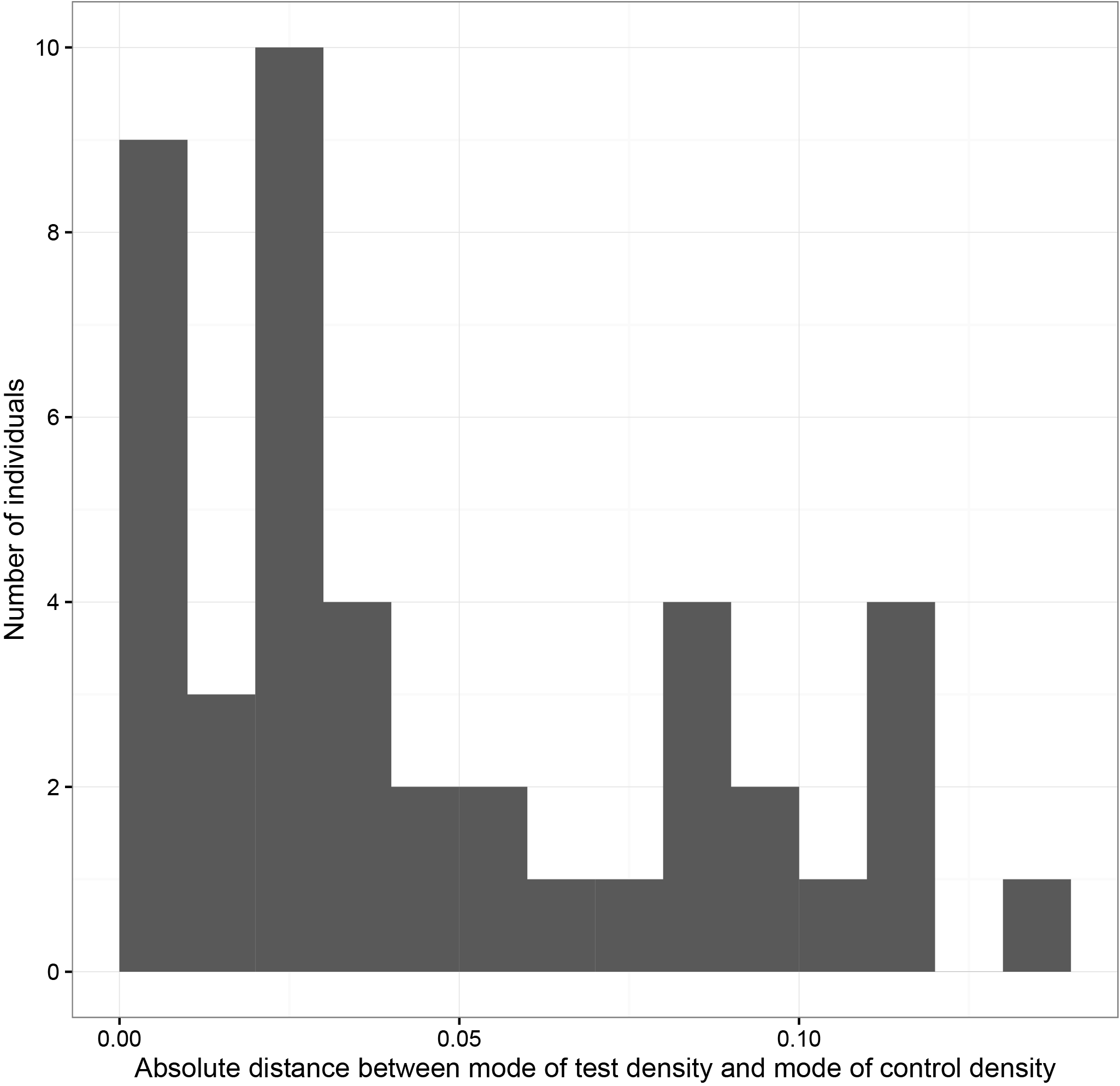

Supplement: Supplementary file 4 — Figure S2. Histogram of the number of calls passing local FDR threshold of 0.05 using our control data-based method. (PNG 35 kb) [file 12859_2018_2356_MOESM4_ESM.png]
